# Use of pharmacogenomics in pediatric renal transplant recipients

**DOI:** 10.3389/fgene.2015.00041

**Published:** 2015-02-18

**Authors:** Mara Medeiros, Gilberto Castañeda-Hernández, Colin J. D. Ross, Bruce C. Carleton

**Affiliations:** ^1^Laboratorio de Investigación en Nefrología y Metabolismo Mineral, Hospital Infantil de México Federico GómezMéxico, México; ^2^Departamento de Farmacología, Facultad de Medicina UNAMMéxico, México; ^3^Pharmaceutical Outcomes Programme, Pediatrics, BC Children's Hospital, University of British ColumbiaVancouver, BC, Canada; ^4^Departamento de Farmacología, Centro de Investigacion y Estudios Avanzados del Instituto Politecnico NacionalMéxico, México; ^5^Division of Translational Therapeutics, Department of Paediatrics, Faculty of Medicine, University of British ColumbiaVancouver, BC, Canada; ^6^Child and Family Research Institute, University of British ColumbiaVancouver, BC, Canada

**Keywords:** pharmacogenomics, genotype, adverse reactions, immunosuppression, drug metabolism, acute rejection, graft survival, GWAS

## Abstract

Transplant recipients receive potent immunosuppressive drugs in order to prevent graft rejection. Therapeutic drug monitoring is the current approach to guide the dosing of calcineurin inhibitors, mammalian target of rapamycin inhibitors (mTORi) and mofetil mycophenolate. Target concentrations used in pediatric patients are extrapolated from adult studies. Gene polymorphisms in metabolizing enzymes and drug transporters such as cytochromes CYP3A4 and CYP3A5, UDP-glucuronosyl transferase, and P-glycoprotein are known to influence the pharmacokinetics and dose requirements of immunosuppressants. The implications of pharmacogenomics in this patient population is discussed. Genetic information can help with achieving target concentrations in the early post-transplant period, avoiding adverse drug reactions and drug-drug interactions. Evidence about genetic studies and transplant outcomes is revised.

## Renal transplantation in children

Renal transplant is a rational therapeutic option for children with end stage renal disease (Dharnidharka et al., 2014). The success of transplant has improved in the past decades, mainly due to the use of potent immunosuppressive regimes to prevent graft rejection (Hartono et al., 2013). From 1980 to 2012 the first year acute rejection rate has declined from 80 to 10–15% (Dharnidharka et al., 2014).

As depicted in Table 1, there are several differences between pediatric and adult renal transplant recipients. The leading cause of kidney failure in children is congenital abnormalities of the kidney and urinary tract (CAKUT) whereas in adults the main causes are diabetes and hypertension. Consequently, perioperative and long-term care differ between children and adults. Age-related changes in the immune system can impact antigen presentation and immune response to the allograft. For example, variation in thymic response is especially important in young children, as the thymus undergoes involution with advancing age (Linton and Dorshkind, 2004; Dorshkind and Swain, 2009). Moreover, T cells are naïve in children, and have reduced expression of CD40L (Brugnoni et al., 1996). CD4+ T cells subsets are primarily defined as their ability to produce interferon gamma (IFN-γ) and IL4 as T helper 1 (Th1) or Th2, in a study evaluating age dependant CD4+ T cell responses upon stimulation with staphylococcal enterotoxin, it was noted that the frequency of activated T cells that produce IFN-γ remained lower than adults until 10 years of age, meaning a tendency for Th2 response (Dorshkind et al., 2009; Hanna-Wakim et al., 2009). Age-related changes in pharmacokinetics have been described, as the absorption, distribution, metabolism and excretion of drugs change with age. Infants generally have a higher metabolic capacity and may require higher doses or reduced dosing intervals for many drugs, and the toxicity profile can also be different (Kearns et al., 2003; Sage et al., 2014). A major hepatic enzyme involved in drug biotransformation at birth is CYP3A7 and its expression declines during the first 6 months of life, whereas CYP3A4 reaches adult levels after 1 year of age (Hines, 2008). In addition, the risk of viral infectious disease and related complications post-transplant change with age, since young children are less likely to be exposed to certain viruses such as Epstein-Barr virus, Cytomegalovirus and BK virus, and if acquiring a primary infection after transplant, the outcome can be worse (Dharnidharka et al., 2014). Comparing 10-year transplant survival by age, young children have the best 10-year graft survival with renal transplantation, while adolescents and elderly people have the worst rate of survival (Scientific Registry of Transplant Recipients, 2012). The dose and therapeutic drug monitoring target concentrations for immunosuppressants are extrapolated from the information obtained in adults, and therefore most of these drugs are used without adequate validation in children despite decades of use for some of these drugs, such as azathioprine (Frattarelli et al., 2014). Finally, not all immunosuppressants have liquid oral or microgranule formulations suitable for young children. Incorrect compounding of acceptable oral formulations for this age group, from adult drug formulations, is a common source of error (Ali et al., 2014).

**Table 1:** **General differences between adult and pediatric renal transplantation**.

	**Adults**	**Children**	**Implication**
Main causes of end stage renal disease	Diabetes and hypertension	CAKUT and glomerulopathies	Children may require urological surgery or vesical cathetherism after transplantation. Some glomerulopathies such as focal segmental glomerulosclerosis have a high rate of relapse after transplantation
Immune system	Thymic atrophy	Thymic output is robust	Antigen presentation, T cell proliferation and immune damage response change with age. This can explain the better results of graft survival in children younger than 5 years
	Higher expression of CD40L on T cells	Low expression of CD40L on T cells	
		Reduced T cell effector function	
		Higher percentage of tolerogenic dendritic cells	
Drug Pharmacokinetics and access to new drugs	Pharmacokinetics is well characterized in adult subjects before marketing.	Developmental changes in absorption, distribution, metabolism and excretion Drugs are often prescribed off-label.	Young children in general require higher doses in mg/kg than adults to achieve the same therapeutic drug levels.
	New drugs can be prescribed soon after approval	Dose and therapeutic drug monitoring are extrapolated from adults	Metabolites, adverse reactions and toxicities may be different with different age groups, even within childhood
Viral infection risk	CMV, EBV and BKV seropositive recipients are more common	Children might be seronegative to many viral agents	Primary infection after transplant can make the outcome worse (i.e., graft or patient loss, post-transplant lymphoproliferative disease)
Living donor 10 year graft survival	65% in recipients 35–49 years	80% in recipients <5 years	Adolescents need enhanced support for therapeutic adherence
	40% in recipients >65 years	50% in recipients 12–17 years	
Drug formulations of immunosuppressants	Easy to administer, enteric coated and extended release formulations available	Liquid oral or microgranule formulations are not available for all immunosuppressants	Compounding from adult formulations is needed, which can lead to serious errors in dosing

## Pharmacogenomics in renal transplant

Pharmacogenomics offers an additional means of further individualizing drug therapy by incorporating genetic information to help guide safer and more effective treatment decisions. Pharmacogenomics aims to integrate an individual's genetic variability with the pathophysiology of disease, pharmacokinetic drug disposition, and predicted disease outcomes to help aid in the prediction of therapeutic efficacy and likelihood of adverse drug reactions (Rieder and Carleton, 2014).

With regards to renal transplantation, the genetics of both the recipient and the donor can be important for both graft and patient outcome (Table 2). The best example in organ transplantation is HLA matching between donor and recipient. Improved HLA matching has increased graft and patient survival, reduced the need for immunosuppression, and reduced the risk of infections and death with functioning grafts (Opelz and Dohler, 2012; Susal and Opelz, 2013). In children, the presence of two HLA DR mismatches increases the risk of non-Hodgkin lymphoma by two-fold [hazard ratio (HR) of 2.04 [95% confidence interval (CI) 1.11–3.74] *p* = 0.021] (Opelz and Dohler, 2010a). In adults, HLA-DR mismatches are also associated to non-Hodgkin lymphoma (HR for two mismatches = 1.56 [95% CI 1.21–1.99], *p* < 0.0001) (Opelz and Dohler, 2010b) as well as osteoporosis and hip fractures (two mismatches HR for hip fracture 2.24 [95% CI 1.25–4.02], *p* = 0.007) (Opelz and Dohler, 2011).

**Table 2:** **Immunosuppressive drugs used in pediatric transplantation and gene polymorphisms related to their effects, metabolism, or transport (Data from organ transplant/kidney diseases studies only)**.

**Drug**	**Description**	**Gene/Protein**	**Variant**	**Effect**	**Study characteristics**	**References**
Corticosteroids	Anti-inflammatory, immunosuppressant	*NR3C1*/glucocorticoid receptor	rs41423247	Increased glucocorticoid sensitivity	95 Japanese renal transplant adults	Miura et al., 2009
				CC allele is associated with lower prednisolone		
				Cmax/Dose (*p* = 0.021) and lower AUC (*p* = 0.029)		
			GR-9β haplotype carriers	Increased incidence of relapse and steroid dependence in children with nephrotic syndrome	113 children with nephrotic syndrome.	Teeninga et al., 2014
				Carriers had a higher incidence of steroid dependence (HR 3.05 [95% CI 1.37–6.74] *p* = 0.006) and frequent relapses (HR 1.98 [95% CI 1.05–3.75], *p* = 0.036)		
Azathioprine	Anti-proliferative	*TPMT*/Thiopurine methyltransferase	rs1800642	Life-threatening myelosuppression	157 adult transplant recipients with TPMT deficiency	Kurzawski et al., 2009
			rs1800460	Associated alleles predicted leukopenia (OR 4.26 [95%CI 1.49–12.1], *p* = 0.008)		
			rs1142345			
			rs56161402			
Mofetil Mycophenolate	Anti-proliferative	*CYP2C8/*Cytochrome P450 2C8	rs11572076	Higher risk of anemia, (Hazard ratio 3.24, 95% CI 1.7–6.2, *p* = 0.00037)	978 adult renal transplant recipients	Jacobson et al., 2011
				Lower risk of leukopenia in carriers of the wild type genotype (GG vs. GA HR 0.14 [95% CI 0.03–0.59], *p* = 0.00783)	189 adult renal transplant	Woillard et al., 2014
		*UGT2B7/*UDP-glucuronosyl-transferase 2B7	rs7438135	Increased risk of anemia in GG vs. AA (HR 1.88 [95%CI 1.23–2.88], *p* = 0.003)	189 adult renal transplant	Woillard et al., 2014
Sirolimus	mTOR inhibitor	*CYP3A4/*Cytochrome p450 3A4	rs35599367	20% lower metabolic rate *in vitro*, but no change in pharmacokinetics	113 adult renal transplant recipients switched from cyclosporine to sirolimus	Woillard et al., 2013
Cyclosporin	Calcineurin inhibitor	*CYP3A4/*Cytochrome p450 3A4	rs2740574	9% higher oral cyclosporine clearance No impact on cyclosporine pharmacokinetics No impact on cyclosporine pharmacokinetics	151 adult kidney and heart transplant recipients	Hesselink et al., 2004
		rs776746,	*CYP3A5 ABCB1/*p-glycoprotein			
		rs10264272				
		rs1045642				
		*ABCB1/*p-glycoprotein	rs2032582	1.3–1.6 higher cyclosporine bioavailability and lower pre-hepatic extraction ratio, only in subjects >8 years	104 pediatric renal transplant recipients	Fanta et al., 2008
			s1128503			
		*XPC/*Xeroderma pigmentosum complementation group C	rs2228001	Cyclosporine nephrotoxicity HR 0.41 [95%CI 0.27–0.63], *p* = 0.00004	323 renal transplant recipients treated with cyclosporine and 692 treated with tacrolimus	Jacobson et al., 2012
		*CYP2C9*/Cytochrome p450 2C9	rs1057910	Cyclosporine nephrotoxicity		
				HR 3.5 [95%CI 1.91–6.61], *p* = 0.00006		
		*PAX4*/paired box gene 4	rs712704	Cyclosporine nephrotoxicity		
				HR 2.09 [95%CI 1.45–3.01], *p* = 0.00007		
Tacrolimus	Calcineurin inhibitor	CYP3A4/Cytochrome p450 3A4	rs3599367	50% lower tacrolimus dose requirements in non-CYP3A5 expressers	129 adult renal transplant recipients (59 tested at 3 months, 80 tested 1–5 years after transplantation)	de Jonge et al., 2014
		CYP3A5/Cytochrome p450 3A5	rs776746	Tacrolimus chronic toxicity, more frequent in those expressing the enzyme, HR 2.38 [95%CI 1.15–4.92], *p* = 0.01	304 adult renal graft recipients	Kuypers et al., 2010
				Tacrolimus dose requirement is lower in non-CYP3A5 expressers	291 renal transplant recipients (124 adults, 167 pediatric)	Garcia-Roca et al., 2012

### Immunosuppressive therapy

Immunosuppressants used in pediatric renal transplantation are listed in Table 2, as well the gene variants related to their therapeutic effects, metabolism and transport. Many of these drugs exhibit a narrow therapeutic index, where even small differences in dose or blood concentration may lead to serious therapeutic failures and/or adverse reactions. The most common long-term combination regimen consists of steroids, tacrolimus, and mofetil mycophenolate (Scientific Registry of Transplant Recipients, 2012).

### Glucocorticoids

Synthetic glucocorticoids are used as potent anti-inflammatory and immunosuppressive agents. Part of the variability in the response to steroids is due to genetic variation in the glucocorticoid signaling pathways, particularly the glucocorticoid receptor, encoded by the *NR3C1* gene and its splice variants (Koper et al., 2014). Several genetic variations in *NR3C1* have been associated with increased glucocorticoid sensitivity (rs41423247, rs6195), and decreased sensitivity (rs6189, rs6190, rs6198). Specifically, the C/C genotype of rs41423247 is associated with a significantly lower prednisolone drug bioavailability in Japanese renal transplant recipients (Miura et al., 2009). Moreover, recently it was found that pediatric patients with nephrotic syndrome who carry the beta haplotype variant in the glucocorticoid receptor exhibit a significantly worse outcome, with increased frequency of first relapse, higher frequency of relapses and steroid dependence (Teeninga et al., 2014). Even though there have been significant efforts to withdraw steroids or even to avoid steroids altogether in renal transplantation (Grenda, 2013), approximately 60% of pediatric transplant patients continue to receive steroids (Dharnidharka et al., 2014). The most common glucocorticoid adverse effects include cushingoid appearance, dyslipidemias, diabetes mellitus, cataracts, hypertension, growth impairment, osteoporosis, femoral avascular necrosis, cataracts, impaired healing and increased susceptibility to infection (Tredger et al., 2006).

#### Calcineurin inhibitors

Cyclosporine and tacrolimus are potent immunosuppressants that inhibit calcineurin, a protein phosphatase involved in T cell activation. Much of the success of renal transplantation has been due to the introduction of cyclosporine in 1983. Tacrolimus has now largely replaced cyclosporine in the treatment of transplant patients, due to its enhanced potency and fewer cosmetic adverse effects. Both share adverse events such as acute and chronic nephrotoxicity (Issa et al., 2013), hypertension, dyslipidemia, and increased risk of new onset diabetes mellitus (Heisel et al., 2004). Cyclosporine also produces hirsutism and gingival hyperplasia (Halloran, 2004).

Cyclosporine and tacrolimus metabolism depend largely upon the liver and intestine expression of the phase I metabolizing enzymes CYP3A4 and CYP3A5 in the liver and intestine. These drugs are known to be transported across cell membranes by p-glycoprotein (Table 2).

Hesselink and coworkers studied the impact of CYP3A4^*^1B (rs2740574), CYP3A5^*^3 and 6 (rs776746, rs10264272), and *ABCB1* (rs1045642) on cyclosporine pharmacokinetics in 151 adult kidney and heart transplant recipients. Patients carrying the CYP3A4^*^1B variant allele exhibited a 9% increased clearance of oral cyclosporine; while none of the other studied variants influenced cyclosporine pharmacokinetics (Hesselink et al., 2004). In a study of 104 pediatric renal transplant recipients (0.36–16.3 years), Fanta et al, examined the potential role of genetic variations in *ABCB1*, *CYP3A4*, *CYP3A5*, and *SLCO1B1* in cyclosporine pharmacokinetics. Interestingly, genetic factors were found to affect the bioavailability only in patients older than 8 years of age, specifically those carrying the *ABCB1* variants rs1128503, rs2032582, had 1.3–1.6 higher cyclosporine bioavailability and lower pre-hepatic extraction ratio than non-carriers, but no significant differences were observed in subjects younger than 8 years. The authors suggest that developmental changes may confound the effect of genetic factors, therefore questioning the value of pharmacogenetic testing in young children (Fanta et al., 2008). Further studies in young children will help to better define the role of ontogeny on the utility of these pharmacogenetic biomarkers.

For tacrolimus, the *CYP3A5* gene polymorphism rs776746 has been associated with lower dose requirements and bioavailability in both adults and children. This variant causes a loss of CYP3A5 activity due to a splice site variant leading to a truncated, inactive enzyme. Interestingly, this association appears to be independent of the tacrolimus formulation (Jacobo-Cabral et al., 2014). The allelic frequency changes with ethnicity, 70–80% of African Americans express the enzyme, in contrast to 5–15% of Caucasians, 30% of Asians and 50% of Mexicans (Garcia-Roca et al., 2012). Nevertheless, the variant has not been associated with acute rejection, probably because transplant patients end-up achieving target concentrations of tacrolimus thanks to therapeutic drug monitoring and close monitoring overall (Hesselink et al., 2008).

In a prospective study, adult patients were randomized to receive tacrolimus either according to standard protocol, or based on *CYP3A5* genotype to guide dosing decisions. Dosing by genotype acquired tacrolimus target concentrations 75% more rapidly and with fewer dose modifications. On day 3 of tacrolimus therapy, 43% of those patients whose therapy was guided by genotype had reached target trough concentrations in contrast to 29% who received standard treatment (Thervet et al., 2010). Nevertheless, the impact of obtaining target tacrolimus concentrations sooner on longitudinal outcomes such as graft survival or rejection episodes has not been reported. Kuypers et al. reported that renal transplant patients expressing CYP3A5 who require higher doses to achieve therapeutic trough concentrations have a higher risk of chronic nephrotoxicity (HR 2.38 [95% CI 1.15–4.92]) (Kuypers et al., 2010).

Jacobson et al. studied early nephrotoxicity during the first 6 months post-transplant in 323 renal recipients treated with cyclosporine or tacrolimus. Nephrotoxicity was defined as a rise in serum creatinine that responded to the reduction or discontinuation of the calcineurin inhibitor. Nephrotoxicity was observed in 22.6% of cyclosporine and 19.8% of tacrolimus treated patients respectively. Interestingly, gene polymorphisms in *XNC, CYP2C9* and *PAX4* were associated with cyclosporine nephrotoxicity, but not with tacrolimus toxicity (Table 2) (Jacobson et al., 2012).

Residual variability can also be attributed to other gene polymorphisms, such as CYP3A4^*^22 (rs35599367) that reduces CYP3A4 activity by 20% (Elens et al., 2011), and leads to 50% lower tacrolimus dose requirements in patients with concurrent non-expression of CYP3A5 (de Jonge et al., 2014).

In addition, the role of ABCB1 remains unclear. There are controversial studies about the impact of *ABCB1* (p-glycoprotein) genes on the pharmacokinetics of tacrolimus, due to the importance of the transporter in the absorption, distribution and elimination of drugs, but its overall effect on tacrolimus bioavailability appears to be small (Mai et al., 2004; Hesselink et al., 2014).

#### Antiproliferatives

***Azathioprine***. The only immunosuppressant used in transplantation that has an FDA-approved drug label with pharmacogenetic information is azathioprine. Nevertheless, its use has declined over the past decade due to the substitution of mycophenolate mophetil in post transplant immunosuppression regimens. Azathioprine is still used in patients who exhibit severe adverse effects from mycophenolate mofetil, and in some countries where the price of mycophenolate mofetil is prohibitive (Bansal et al., 2011). Azathioprine is a prodrug, rapidly converted to 6-mercaptopurine, which is further converted cytotoxic thioguanine nucleotides by hypoxanthine-guanine phosphoribosyltransferase and a series of enzymatic process including kinases. The thioguanine nucleotides reduce inflammation by inhibiting DNA replication and blocking CD28 costimulatory signaling via RAC1. Excess thioguanine nucleotides induce myelosupression and are inactivated by the enzyme thiopurine methyltransferase (TPMT) to methylcaptopurine nucleotide. To date, 37 variants in TPMT alleles have been identified (Roberts et al., 2014). The polymorphisms in TPMT that affect azathiopurine toxicity are related to the activity of the enzyme. Approximately 0.3 to 1% of patients of European ancestry inherit two non-functional TPMT alleles (homozygous) for low or absent TMPT activity (also called poor methylators), and 10% inherit a single non-functional allele and exhibit intermediate enzyme activity and have 90% normal activity. Poor methylators are at risk of developing severe life-threatening myelotoxicity if they receive azathioprine (Weinshilboum and Sladek, 1980). Therefore, genotyping or phenotyping by measuring TPMT activity is recommended to identify patients at risk. Nearly 95% of poor TPMT methylation is due to four alleles TMPT^*^2 (238 G>C, rs1800462), TMPT3^*^A (460G>A rs1800460; 719 A>G rs1142345), TPMT^*^3C (719 A>G rs1142345) and TMPT^*^8 (644G>A, rs56161402). There is significant interethnic variability in the frequency of these alleles (Roberts et al., 2014). Kurzawski et al evaluated the most common variants in 157 renal transplant recipients, and variant carriers had higher risk of leukopenia (OR 4.26 [95% CI 1.49–12.1], *p* = 0.008) (Kurzawski et al., 2009).

***Mycophenolic acid***. Mycophenolic acid is an inhibitor of inosine 5-monophosphate dehydrogenase (IMPDH), a key enzyme responsible for the *de novo* pathway of guanosine nucleotide synthesis in B and T lymphocytes. Inhibition of IMPDH thus us reduces cell proliferation and suppresses antibody formation and cell-mediated immune responses, which are key factors in graft rejection. Mycophenolic acid was approved by the FDA to prevent transplant rejection in 1995. For children, a pediatric oral solution is available in some countries. Mycophenolate mofetil is a prodrug (2, 4- morpholinoethylester of mycophenolic acid) that is hydrolyzed by esterases in gastrointestinal tract, blood and liver into the active drug mycophenolic acid. This is subsequently metabolized by the uridine diphosphate-glucuronosyltransferase enzymes (UGT), primarily UGT1A9 in the liver and UGT1A8 in the intestine to a stable, inactive phenolic glucuronide. The UGT2B7 produces a mycophenolate-acyl-glucoronide but the effect on inhibition of IMPDH is minimal compared to mycophenolic acid (Gensburger et al., 2009). Adverse effects include anemia, leukopenia and gastrointestinal adverse effects that can lead to dose reduction and low drug exposure concentrations with the consequent risk of acute rejection episodes or graft failure. An enteric-coated formulation of mycophenolic acid is available as mycophenolate sodium, and conversion to equimolar doses of mycophenolate mofetil to enteric-coated mycophenolic acid leads to similar bioavalability in children (Reyes et al., 2010). There is a high int-ra and interpatient variability in the bioavailability of mycophenolic acid, and since it undergoes enterohepatic recirculation, more than one peak can be observed after oral administration (Filler, 2006; Reyes et al., 2010).

In a study including 978 adult renal transplant recipients treated with mycophenolate mofetil, anemia occurred in 9.5% and leukopenia in 22.9% of patients. *CYP2C8* rs11572076 was associated with the risk of anemia (HR 3.4 [95% CI 1.7–6.20], *p* = 00037), as was *IL12A* rs568408 (HR 1.98 [95% CI 1.39–2.82], *p* = 0.00014) and *HUS1* rs2037483 (HR 0.54 [95% CI 0.39–0.74], *p* = 0.00016). These variants were not associated with the risk of leukopenia (Jacobson et al., 2011). A recent study by Woillard et al in 189 renal transplant adults treated with enteric-coated mycophenolic acid, in whom 15 SNPs were studied (in *IMPDH1*, *IMPDH2*, *ABCC2*, *SLCO1B3*, *CYP2C8*, *HUS1*, *UGT1A8*, *UGT1A9*, *UGT2B27*, *IL12A*), the variant allele *UGT2B7* (840 G>A rs7438135) was associated with an increased risk of anemia (GG vs. AA HR 1.8 [95% CI 1.23–2.88], *p* = 0.0034). In the same study, *CYP2C8* rs11572076 wild type was associated with a lower risk of leukopenia (HR 0.14 [95% CI 0.03–0.59], *p* = 0.0078), but not with anemia. The authors did not find any of the studied variants associated with diarrhea or biopsy-proven acute rejection (Woillard et al., 2014).

***mTOR inhibitors***. Sirolimus (also known as rapamycin) is an inhibitor of mammalian target of rapamycin (mTOR) and the activation of the cell cycle. Specifically, it prevents the generation of the coestimulatory signal during G_0_–G_1_ in lymphocytes, and the post-transduction events after cytokine stimulation of G_1_ (Stenton et al., 2005). This drug was approved by the FDA in 1999 to prevent acute rejection in renal transplant recipients. It is metabolized by CYP3A and it is also a substrate for p-glycoprotein. Liquid and solid oral formulations are available. As with other immunosuppressants, it exhibits a high intra- and inter-patient variability in pharmacokinetics, and therapeutic drug monitoring is recommended. Adverse effects include hyperlipidemia, thromocytopenia, peripheral edema, dermatitis and impaired wound healing (Halloran, 2004; Tredger et al., 2006).

There are controversial results in studies about the relevance of gene polymorphisms in *CYP3A* and *ABCB1* (p-glycoprotein), pharmacokinetics and outcome. Woillard et al. studied 113 renal transplant adults switched from a calcineurin inhibitor to sirolimus. They genotyped patients for CYP3A4^*^22 (rs35599367), CYP3A5^*^3 (rs776746), *POR* (p450 oxidoreductase, rs1057868), and *PPARA* (Peroxisome proliferator activated receptor alpha, rs4253728). Patients with CYP3A4^*^22 had a 20% lower sirolimus metabolic rate *in vitro*, but no differences were observed in the pharmacokinetics or the frequency of adverse effects (Woillard et al., 2013).

## Recipient genotype and risk of acute rejection

The progress in the field of pediatric transplantation has reduced the 1-year acute rejection rate from 55% in late 1980's to 15% today (Dharnidharka et al., 2014). Even when successfully treated, acute rejection reduces the survival rate of the graft (Rusai and Szabo, 2014).

There are controversies regarding the association between gene polymorphisms and acute rejection. It has been postulated that the basal production of cytokines, probably determined genetically can be related to the risk of rejection (Goldfarb-Rumyantzev and Naiman, 2010), nevertheless most of the studies have been performed in small sample populations, not corrected for multiple testing, and importantly, without replication in an independent cohort (Phelan et al., 2014).

Recently, a study reported significant associations with variants in the toll-like receptor gene *TLR4* (rs498670, rs4986791). In a cohort of 238 renal transplant recipients, those patients with the *TLR4* variants were less likely to have acute rejection (RR 0.41, [95% CI 0.21–0.93], *p* = 0.001) but more prone to severe bacterial infections (RR 1.33, [95% CI 1.12–1.67], *p* = 0.01) (Ducloux et al., 2005). The authors postulated that since TLR4 pathway is crucial for alloreactive responses and can be activated by endogenous ligands and pathogen associated recognition patterns, the determination of TLR4 genotype could be used to modulate de immunosuppressive treatment and optimize the monitoring and prophylaxis of post-transplant infections.

Chen and coworkers studied the genetic polymorphisms in IL-2 (*rs* 2069762), IL-10 (−592 C>A and −1082 G>A), TGF-β 1 (915 G>C) and IL2-RB (rs228942 and rs228953) in 325 renal transplant recipients 1 year after surgery. However, these variants did not exhibit significant associations with acute rejection (Chen et al., 2014).

To the best of our knowledge, there are no pediatric studies looking at genetic factors that contribute to the risk of acute rejection.

## Donor genotype and transplant outcomes

The *ABCB1*donor genotype rs1045642 has been associated with long-term graft survival, as reported by Moore et al in a discovery cohort of 811 adult transplant recipients (HR 1.69, [95% CI 1.2–2.40], *p* = 0.003), and further validated in 675 donors from Belfast (HR 1.68, [95% CI 1.21–2.32], *p* = 0.002) and in 2985 donors from a Collaborative Transplant Study (HR 1.84, [95% CI 1.08–3.13], *p* = 0.006), since the variant is related to p-glycoprotein expression it might play a role in the development of calcineurin inhibitor nephrotoxicity and fibrosis (Moore et al., 2012).

Apolipoprotein gene (*APOL1*) variants rs73885319 and rs71785313 have been associated with kidney disease while conferring protection against *Trypanosoma brucei rhodesiense*. African Americans with the risk variants have higher odds of developing end stage renal disease due to focal segmental glomeruloesclerosis (OR 10.5, 95% CI 6.0–18.4) or hypertension (OR 7.3, 95% CI 5.6–9.5) (Genovese et al., 2010). In regard to transplant patients, one study with a mean follow up of 26.4 months suggested that kidneys from deceased donors with two *APOL1* risk variants had significantly shorter graft survival (HR 3.84, *p* = 0.008) (Reeves-Daniel et al., 2011) whereas in the case of the receptors, carrying two risk variants alleles do not increase the risk of 5 year allograft loss (HR 0.96, [95% CI 0.61–1.48], *p* = 0.84).

## New technologies

There are few reports using genome wide association studies (GWAS) in renal transplantation. O' Brian et al. recently examined the genetic factors associated with graft function at 5 and 10 years post-transplant in a GWAS of 326 adult Irish donor transplant recipients. After correcting for multiple testing, two variants were significantly associated with 5-year creatinine levels, including rs6565887, a non-coding intronic variant in the zinc finger protein 516 gene (*ZNF516*), which is expressed mainly in lungs, spleen and kidney (*p* = 4.04e-08); and rs3811321, in a non-coding exon of the T-cell receptor alpha chain gene (*TCRA*) (*p* = 7.63 × 10e-08). Together these variants accounted for up to 17.4% of trait variance. These variants were predictors of 10-year allograft survival (rs3811321: 70% vs. 30%, incidence ratio 2.4 *p* = 0.004, rs6565887: 70% vs. 45%, incidence ratio 1.7, and *p* = 0.025). Importantly, these findings still need independent replication (O'Brien et al., 2013).

GWAS was recently used to identify genetic variants related to new onset diabetes after transplantation in adult renal transplant recipients. From a study of 26 cases and 230 controls, 26 SNPs were associated with new onset diabetes. These associations were replicated for eight of the SNPs by follow-up genotyping of a second cohort of patients, 57 cases and 383 controls. These variants included *ATP5F1P6* rs10484821 encoding an inferred mitochondrial ATP synthase pseudogene (OR 3.5 [95% CI 2.1–5.8], *p* = 1.5 × 10^−7^); *DNAJC16* rs7533125 whose transcript is part of the heat shock protein 40 complex (OR 2.4 [95% CI 1.5–3.6], *p* = 0.001), *CELA2B* rs2861484 encoding a pancreatic chymotrypsin-like elastase (OR 2.4 [95% CI 1.5–3.7], *p* = 0.0001); *AGMAT* rs11580170 encoding agmantine, a protein scavenger of reactive oxygen species to protect the mitochondrial membrane (OR 2.2 [95% CI 1.4–3.4], *p* < 0.00), *CASP9* rs2020902 in the caspase 9 gene (OR 2.3 [95% CI 1.5–3.6], *p* < 0.001); *NOX4* rs1836882 that encodes an enzyme of the NAPDH oxidase complex (OR 2.7 [95% CI 1.5–4.8], *p* = 0.001) and *NPPA* rs198372, natriuretic peptide precursor A (OR 2.5 [95% CI 1.5–4.2], *p* < 0.001) (McCaughan et al., 2014).

It has been pointed out that outcomes such as patient and graft survival are complex and likely incorporate the effects of many genes. Studies need to be sufficiently powered to avoid type II errors (false negatives), since significant associations are difficult to demonstrate if the sample size is too small or the SNP is rare in the studied population (Stegall et al., 2014).

Recently, the use of mRNA gene expression analysis of urinary cells has been proposed as a non-invasive and sensitive method to diagnose acute rejection. The use of a three gene molecular signature (IP10, CD3ε and 18s rRNA) was able to discriminate between rejecting and non-rejecting samples (area under the curve [AUC] of the receiver operating curve analysis [ROC] of 0.85, 95% CI 0.78–0.91, *p* < 0.001) (Suthanthiran et al., 2013).

The peripheral blood gene expression by quantitative real-time PCR was used to diagnose acute transplant rejection using a 17-gene set (kidney solid organ response test, kSORT). The study was validated in both adult and pediatric recipients, and accurately indicated the risk and prevalence of acute rejection in 558 transplant recipient samples (final AUC 0.92, 95% CI 0.86–0.98). Further studies are needed to replicate this finding and to determine if kSORT can be used serially post-transplant to stratify patient immune risk, adjust medications, or decide the requirement for biopsy (Roedder et al., 2014).

There is a need for pharmacogenomics studies in children, since ontogeny may modify the relationship between genotype-phenotype including the profile of adverse events (Hines, 2008).

### Conflict of interest statement

The authors declare that the research was conducted in the absence of any commercial or financial relationships that could be construed as a potential conflict of interest.
